# Cellular Signal Transduction Pathways Involved in Acute Lung Injury Induced by Intestinal Ischemia-Reperfusion

**DOI:** 10.1155/2021/9985701

**Published:** 2021-06-04

**Authors:** Guangyao Li, Yingyi Zhang, Zhe Fan

**Affiliations:** Department of General Surgery, The Third People's Hospital of Dalian, Dalian Medical University, Dalian, China

## Abstract

Intestinal ischemia-reperfusion (II/R) injury is a common type of tissue and organ injury, secondary to intestinal and mesenteric vascular diseases. II/R is characterized by a high incidence rate and mortality. In the II/R process, intestinal barrier function is impaired and bacterial translocation leads to excessive reactive oxygen species, inflammatory cytokine release, and even apoptosis. A large number of inflammatory mediators and oxidative factors are released into the circulation, leading to severe systemic inflammation and multiple organ failure of the lung, liver, and kidney. Acute lung injury (ALI) is the most common complication, which gradually develops into acute respiratory distress syndrome and is the main cause of its high mortality. This review summarizes the signal transduction pathways and key molecules in the pathophysiological process of ALI induced by II/R injury and provides a new therapeutic basis for further exploration of the molecular mechanisms of ALI induced by II/R injury. In particular, this article will focus on the biomarkers involved in II/R-induced ALI.

## 1. Introduction

Intestinal ischemia-reperfusion (II/R) injury can occur in a variety of pathophysiological conditions, including acute mesenteric ischemia, severe trauma, acute shock, small bowel transplantation, and sepsis [1]. II/R is a clinical state. During ischemia, the blood supply of the intestine is limited, and then, the tissues and organs are damaged due to reperfusion and oxygen recovery [2]. The recovery of blood flow and oxygen during reperfusion leads to bacterial translocation, tissue damage, inflammatory response, and oxidative stress. During ischemia, tissue hypoxia leads to endothelial cell barrier function damage and increases in vascular permeability, followed by cell death, tissue damage, and organ failure during reperfusion [3]. In addition to intestinal injury, II/R can also lead to distal tissue injury and distal organ failure. Distant organs, especially the lungs, are very sensitive to II/R injury. According to the literature, the mortality of II/R is as high as 60%–80% [4]. An increasing number of reports show that secondary distal organ injury (acute lung injury (ALI) and acute respiratory distress syndrome) is more serious than intestinal injury and has been shown to be the main cause of death in patients with II/R [5].

The pathophysiology and pathogenesis of ALI induced by II/R are complicated and poorly understood. Some researchers have hypothesized that damage to the intestinal mucosal barrier after II/R leads to the translocation of bacteria and endotoxins, which leads to a systemic inflammatory response. The release of a large number of inflammatory mediators (TNF-*α*, IL-1, IL-6, IL-8, IL-10, NO, etc.) into the systemic circulation can lead to cell necrosis, tissue damage, and organ failure. Neutrophils and their products are increased in lung tissue, leading to increased vascular permeability, vascular and pulmonary interstitial edema, and pulmonary edema [6]. However, the specific mechanism of ALI is very complex, involving bacterial translocation, inflammatory response, oxidative stress, and initiation of apoptosis and necrosis. In this review, we focused on the multiple signaling pathways involved in II/R-induced ALI. The search Medical Subject Headings (MeSH) terms and keywords were as follows: acute lung injury, ALI, intestinal ischemia reperfusion, intestinal ischemia-reperfusion, and gut ischemia-reperfusion by using PubMed, Embase, and MEDLINE. Cellular signal transduction pathways such as the MAPK signaling pathway, NF-*κ*B signaling pathway, TLR4 signal transduction pathway, PKC/p66Shc signaling pathway, NLRP3 inflammasome, Nrf2 signaling pathway, SIRT1 signaling pathway, and other signaling pathways were also summarized to explore the potential pathogenesis of II/R-induced ALI and examine new targeted therapies using biomarkers.

## 2. Biomarkers of ALI Induced by Aggravating II/R

### 2.1. MAPK Signaling Pathway

The mitogen activated protein kinase (MAPK) signaling pathway is activated by a variety of inflammatory signals, including inflammatory mediators and oxidative stress factors [7]. At present, four MAPK pathways have been identified in mammals, including extracellular signal-regulated kinase (ERK), c-Jun N-terminal kinase (JNK), p38 mitogen-activated protein kinase, and ERK5 [8]. Different extracellular stimuli can activate different MAPK signaling pathways and mediate different cellular biological responses through their mutual regulation. The ERK signaling pathway plays an important role in the process of cell proliferation mediated by growth factors. JNK and p38MAPK pathways activate downstream factors (AP-1 and Jun), which are related to a variety of pathophysiological processes during cell apoptosis and stress [9]. Together with endoplasmic reticulum stress, IL-1*β* increases apoptosis through the JNK signaling pathway [10]. Recently, the p38MAPK signaling pathway has been proposed to play a key role in the inflammatory response of ALI [11]. During ALI, inflammatory factors enter into the lung tissue and destroy lung endothelial cells, leading to an increase in pulmonary capillary permeability that results in pulmonary edema. Therefore, inflammation and pulmonary edema may be two important pathological features of ALI [12].

It has been reported that one of the most bioactive cytokines in the early stage of ALI is IL-1*β*, which is a powerful inducer of lung inflammation and can cause the release of various proinflammatory factors [13]. The increase in IL-1*β* may be the result of ALI induced by II/R, so reducing the expression of IL-1*β* may be conducive to the recovery from lung injury. Increased expression of p38MAPK can also be observed after II/R. In addition, there are also reports that p38MAPK can activate rat pulmonary interstitial macrophages to produce nuclear factor kappa B (NF-*κ*B) [14]. Studies have shown that the p38MAPK inhibitor sb239063 can effectively reduce the expression level of IL-1*β* after II/R by inhibiting the p38MAPK pathway, significantly improving lung injury, and providing a new therapeutic approach for the clinical application of p38MAPK as an intervention against ALI after II/R [15].

**Figure 1:**
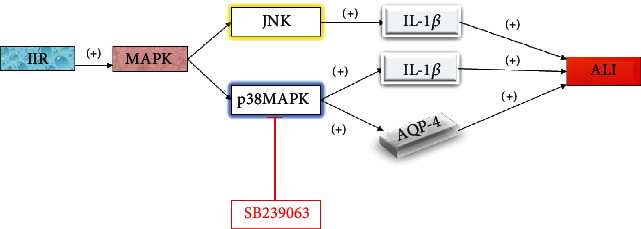
JNK and p38MAPK in the MAPK pathway aggravate ALI, while SB239063 inhibits the p38MAPK pathway to relieve ALI.

Aquaporins (AQPs) are a small family of integral membrane proteins that regulate water transport and play an important role in water homeostasis. Aquaporin 4 (AQP4) is a recently found protein related to edema [16]. Pulmonary edema, as an important pathological feature of ALI caused by II/R, suggests a novel approach for the treatment of ALI. Studies have shown that the increased expression of AQP4 is related to the severity of lung injury induced by II/R, and AQP4 plays an important role in the pathogenesis of ALI induced by II/R. The p38MAPK inhibitor SB239063 reduced the expression of AQP4 and alleviated the extent of lung injury, suggesting that p38MAPK may be the main pathway mediating the expression of AQP4 in ALI induced by II/R. Inhibition of the p38MAPK pathway may thus become an effective target for the prevention and treatment of ALI induced by II/R [17] (Figure 1).

### 2.2. NF-*κ*B Signaling Pathway

NF-*κ*B plays an important role in transcriptional regulation of many inflammatory and apoptotic regulatory genes during II/R. NF-*κ*B is a rapid nuclear transcription factor. At present, there are five members of the NF-*κ*B family in mammalian cells: NF-*κ*B1 (P50), NF-*κ*B2 (p52), Rel A (p65), Rel B, and c-Rel. NF-*κ*B must form as a homodimer or heterodimer to have biological activity. When cells are stimulated by internal and external factors, NF-*κ*B is activated and enters the nucleus to regulate gene transcription, including the genes for TNF-*α*, IL-1, IL-6, IL-8, and ICAM-1. Inflammatory factors play an important role in II/R injury, and TNF-*α*, as the induction factor, induces the release of various inflammatory factors [18–20].

**Figure 2:**
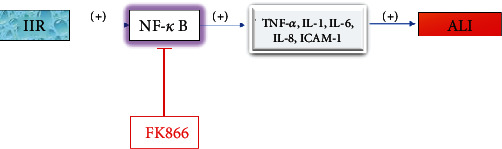
Nuclear translocation of p65 in NF-*κ*B aggravates ALI, while FK866 inhibits p65 nuclear translocation to relieve ALI.

NF-*κ*B enters the nucleus and mediates the transcription and release of a variety of inflammatory factors, which then spread the inflammatory response and are causative factors leading to ALI. Inhibition of the NF-*κ*B pathway can reduce lung inflammation and ALI caused by II/R [21]. After II/R, the levels of visfatin in plasma and lung tissue are significantly increased, and visfatin exerts a proinflammatory effect by upregulating the production of the proinflammatory factors IL-1*β*, IL-6, and TNF-*α* in a dose-dependent manner [22]. The visfatin inhibitor FK866 inhibits the nuclear translocation of NF-*κ*B p65 by inhibiting the degradation of cytoplasmic I-*κ*B*α*. Whether FK866 has the effect of reducing apoptosis in the process of inflammation needs further study. However, it has been reported that TNF-*α*, one of the main mediators of ALI, starts the apoptosis cascade, and FK866 at least partially inhibits apoptosis in ALI through an indirect pathway [23]. FK866 can significantly reduce the inflammatory response and apoptosis after organ injury by inhibiting the NF-*κ*B signaling pathway and ultimately improves the survival rate [22]. Curcumin can also effectively prevent II/R-induced ALI by inhibiting the NF-*κ*B pathway. After curcumin treatment, myeloperoxidase levels in lung tissue (a marker of neutrophil recruitment and lung injury [24]) were significantly decreased, while superoxide dismutase (SOD) level (an indicator of antioxidant effect [25]) was significantly increased. The levels of IL-6 and ICAM-1 were parallel to the changes of NF-*κ*B, suggesting that curcumin can reduce the recruitment/infiltration of neutrophils and play an anti-inflammatory and antioxidant role by inhibiting the NF-*κ*B pathway [26] (Figure 2).

### 2.3. TLR4 Signal Transduction Pathway

Toll-like receptors (TLRs) are a class of important proteins involved in innate immunity and act as the first barrier against infectious diseases. TLR4, a member of the TLR family, is responsible for recognizing pathogens and activating the innate immune system. It can recognize a variety of ligands, such as endogenous ligands (low-density lipoprotein and heat shock protein) and exogenous ligands (lipopolysaccharide), so it plays a key role in the body's response to I/R injury [27]. TLR4 can activate multiple signaling pathways after specifically binding with ligands, including MAPK and NF-*κ*B pathway proteins, which are key factors involved in the inflammatory immune response that regulate cell survival [8]. In the lung injury model induced by II/R in mice, TLR4 deletion can prevent the activation of p38MAPK and NF-*κ*B signals, and the phosphorylation of p38MAPK and the activation of NK-*κ*B in the lung tissue of TLR4-mutant mice are significantly inhibited, which indicates that these pathways are involved in ALI induced by II/R and are mediated by TLR4 [28]. The downstream effects regulated by TLRs vary with the type of receptors, and these mediators contribute to the production of local inflammation and the aggregation of neutrophils [29].

The signal transduction pathway mediated by TLRs depends on the interaction with cytoplasmic adaptor proteins and mainly myeloid differentiation factor 88 (MyD88). MyD88 is considered the central adaptor protein for signal transduction activated by MAPK and NF-*κ*B in almost all TLRs (except TLR3) [30]. In fact, in the absence of MyD88, bacterial translocation is weakened and intestinal and lung injuries are alleviated, which is due to the reduction in neutrophil aggregation, lower levels of inflammatory mediators, alleviation of pulmonary vascular injury, and the improved survival rate [31]. Victoni et al. confirmed that blocking the TLR/MyD88 pathway reduced intestinal and lung injury after II/R, thus improving the survival rate [32].

**Figure 3:**
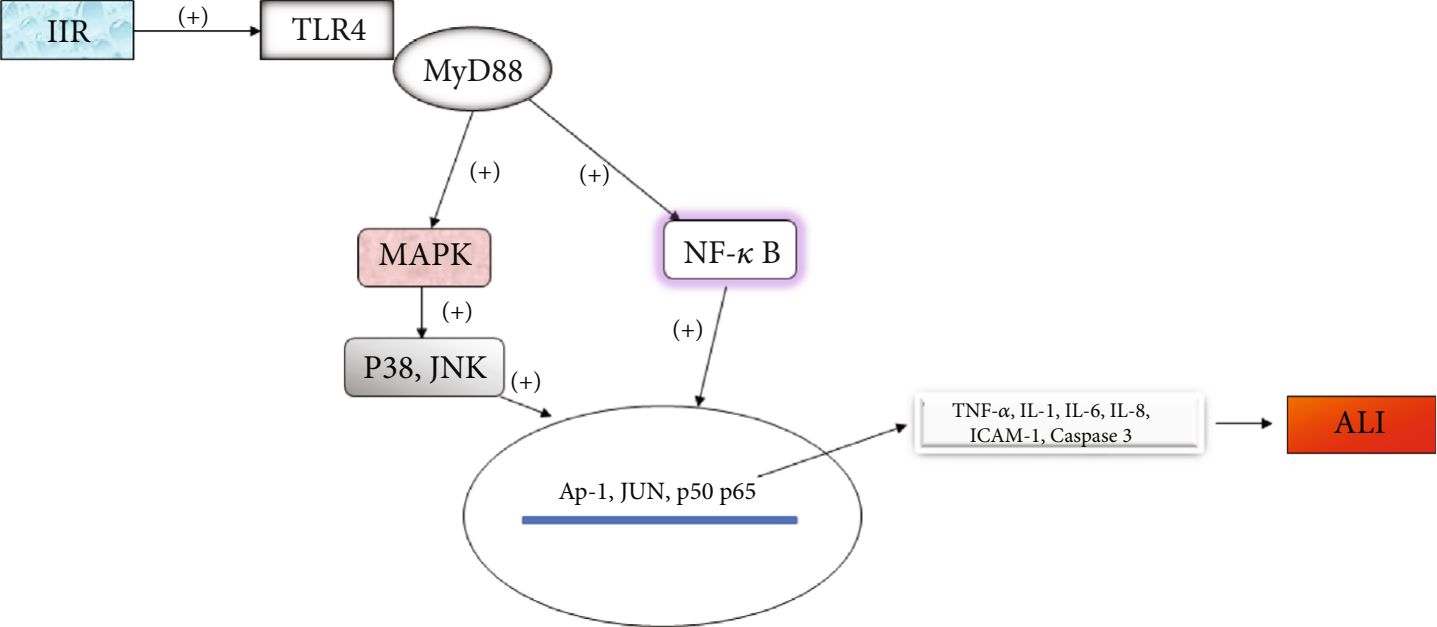
The TLR4 pathway regulates MAPK and NK-*κ*B pathways to aggravate ALI.

TLRs play an important role in innate immunity by regulating the activity of different NF-*κ*Bs. It was reported that the TLR4/NF-*κ*B signaling pathway is the key mechanism regulating proinflammatory factors in the II/R-induced lung injury model [28]. Activation of TLR4 can promote the activation of NF-*κ*Bp65 and lead to the release of proinflammatory factors TNF-*α*, IL-1, IL-6, and IL-8 from pulmonary macrophages, resulting in ALI. The *α*-7 nicotinic acetylcholine receptor agonist can inhibit the expression of TLR4, reduce the transport of p65, diminish the activation of NF-*κ*B and production of proinflammatory factors, and also inhibit the inflammatory reaction, thus reducing ALI caused by II/R [33]. In addition, bone marrow mesenchymal stem cells can downregulate the expression of TLR4/NF-*κ*B and reduce cell apoptosis and inflammatory responses, thereby alleviating ALI induced by II/R. The inactivation of TLR4/NF-*κ*B signaling also leads to the downregulation of caspase-3, a key protease in the apoptotic response [34]. The results of this study suggested that using bone marrow mesenchymal stem cells to target TLR4 offers a therapeutic regimen for ALI induced by II/R. The same results were verified in an experiment by Soares et al. The lung inflammatory response and apoptosis in TLR4-deficient mice were significantly reduced, which confirms that the TLR4 receptor signaling pathway plays an important role in ALI. Consequently, interfering with the TLR signal may be a promising therapeutic strategy [35] (Figure 3).

### 2.4. PKC/p66Shc Signaling Pathway

SHC protein is widely expressed in mammals and is a proapoptotic factor and proinflammatory mediator. Adaptor protein p66Shc (a member of the SHCA protein family) is a proapoptotic protein, which is composed of p64shc and p52shc proteins. Its proapoptotic effect mainly depends on the binding of cytochrome-c in mitochondria and its oxidation function; in the oxidation process, cytochrome-c is released into the cytoplasm to activate caspase-3, thus inducing apoptosis [36]. The stress response phosphorylates serine 36 of p66Shc, which plays an important role in the oxidative stress response and apoptosis [37]. Manganese superoxide dismutase (MnSOD) and Bcl-2 play an important role in the pathophysiological process of II/R, and both of which can be regulated by p66Shc [38]. II/R activated changes in reactive oxygen species accumulation, in which p66Shc phosphorylation of antioxidant factors GSH and MnSOD decreased, while phosphorylation of apoptosis-related factors increased for caspase-3 and decreased for Bcl-2. The use of polyphenol protocatechuic acid (PCA) can significantly reduce II/R-induced ALI by inhibiting p66sshc, increasing lung antioxidant factors, and reducing proapoptotic factors and inflammatory mediators [39]. This is consistent with previous studies in which the inhibition of p66Shc phosphorylation had a protective effect on lung epithelial cell apoptosis during ALI induced by II/R [38]. Inhibition of p66Shc phosphorylation may be a new treatment for ALI induced by II/R.

**Figure 4:**
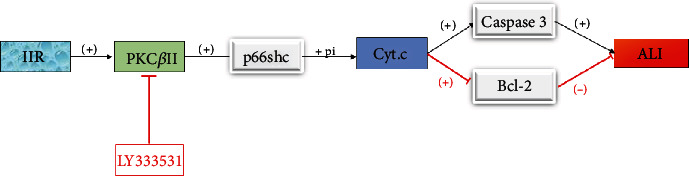
PKC *β*II promotes phosphorylation of p66shc, upregulates caspase-3, and downregulates Bcl-2, thereby aggravating ALI; LY333531 inhibits PKC *β*II and relieves ALI.

Protein kinase C (PKC) plays an important role in signal transduction by phosphorylating serine and threonine residues. Many conditions can cause PKC activation, including II/R [40]. Previous studies have shown that the activation of PKC *β*II specifically participates in the primary injury of II/R, and inhibiting the expression of PKC *β*II can prevent II/R injury [41]. However, PKC *β*II is selectively activated in the lung and liver after II/R. LY333531, a specific inhibitor of PKC *β*II, significantly reduced lung injury, inflammatory response, oxidative stress, and apoptosis after II/R. Meanwhile, it also significantly inhibited the activation of p66Shc and the binding of cytochrome-c, which resulted in a decrease of cytochrome-c release and caspase-3 cleavage, and reduced apoptosis. This study showed that the PKC *β*II/p66Shc pathway could be a specific therapeutic target, which can not only reduce II/R injury but also improve secondary lung injury, providing a new therapeutic strategy for the prevention of ALI caused by II/R [42] (Figure 4).

### 2.5. NLRP3 Inflammasome

**Figure 5:**
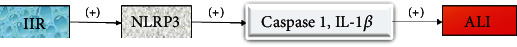
The NLRP3 inflammasome aggravates ALI.

An increasing number of studies have shown that nucleotide-binding oligomerization domain-like receptor (NLR) initiates an inflammatory response in a variety of diseases. When the body is injured, the NLRP3 inflammasome activates caspase-1 and IL-1*β*, leading to inflammation and tissue damage [43]. After II/R, the NLRP3 inflammasome plays an important role in early injury of the heart, liver, kidney, lung, intestine, and other organs [44]. Lipid mediators are effective regulators of innate and acquired immune responses and are associated with many inflammatory diseases [45]. II/R stimulates the release of lipid mediators, which can enhance the expression of NLRP3 inflammatory bodies and the production of IL-1*β* in pulmonary vascular endothelial cells, thereby increasing pulmonary vascular permeability and the inflammatory response and resulting in ALI. Thus, NLRP3 inflammation-driven IL-1*β* is a new potential target for the prevention and treatment of ALI induced by II/R [46] (Figure 5).

### 2.6. mTOR, VAP-1, NADPH Enzyme, IRHOM2, and CIRP

mTOR is a serine/threonine kinase, which plays a key role in cell proliferation and survival. A large number of studies have shown that mTOR plays an important role in the pathogenesis of ALI during II/R [47]. mTOR includes two different compounds, mTORC1 and mTORC2. mTORC1 promotes protein synthesis by phosphorylation of P70S6K and eIF4E binding protein (4EBP) [48]. FKBP25 is a member of the FKBP family of immunoavidin proteins, which can form a complex with mTOR and then plays a role by regulating mTOR [49]. Neurilifordin F (NF) may reduce lung injury by activating FKBP25 and inhibiting the mTOR/P70S6K pathway. On the other hand, NF can reduce the expression of p65 and the activation of IL-1*β* by inhibiting TLR4. The decrease in p65 expression also reduces the activation of NLRP3/caspase-1 and the expression of inflammatory mediators, with an overall anti-inflammatory effect that improves ALI induced by II/R [50]. Similarly, the mTOR inhibitor rapamycin can inhibit the activation of NF-*κ*B and reduce distal lung injury after II/R. Inhibition of the mTOR pathway is now a targeted therapy for ALI after II/R [51].

Leukocyte extravasation is also involved in II/R injury and ALI. Excessive leukocyte extravasation is largely the result of the increased expression of adhesion molecules on the surface of endothelial cells and neutrophils [52]. Vascular adhesion protein-1 (VAP-1) is an extracellular enzyme expressed in endothelial cells, which can regulate leukocyte extravasation. In the II/R state, tissue damage is mainly caused by exudative leukocytes. Blocking adhesion molecules to inhibit the interaction between leukocytes and endothelial cells can reduce the degree of tissue damage in II/R [53]. Jan et al. used gene-targeted animals to show that VAP-1 is important in II/R and ALI. Anti-VAP-1 antibody or small molecule SSAO inhibitor reduced II/R injury and lung injury caused by neutrophil aggregation in the lungs [54].

Oxidative stress is the main underlying factor in ALI induced by II/R, and mast cell activation aggravates oxidative stress and ALI induced by II/R [5]. In the acute lung injury model induced by II/R, NADPH oxidase (p47phox and gp91phox) activity increased. Resveratrol inhibited the activation of mast cells and significantly reduced oxidative stress and inflammatory reactions in lung tissue after IIR [55]. Tryptase released by mast cells also plays a key role in II/R-induced ALI by activating protease-activated receptor-2 (PAR-2). Inhibition of tryptase release may be an effective scheme for the treatment of II/R-induced ALI [5].

TNF-alpha is involved in the pathogenesis of many inflammatory diseases. TNF-alpha converting enzyme (TACE) is necessary for the release of TNF-alpha. Inactivated rhomboid protein 2 (IRHOM2) has recently been identified as an important factor regulating TACE maturation in immune cells. In IRHOM2 gene knockout mice, inflammatory mediators, proapoptotic factors, and lung injury were significantly reduced. Therefore, IRHOM2 may play an important role in the pathogenesis of II/R-induced ALI and may be a new target for the treatment of II/R-induced ALI [56, 57].

The aseptic inflammation during II/R injury is triggered by endogenous injury-associated molecular pattern (DAMP) proteins. Cold-induced RNA-binding protein (CIRP) is a member of the DAMP family and constitutes a new inflammatory mediator, which can cause tissue damage during II/R [58]. DAMP binds to TLRs to enhance the activation of innate immune cells; furthermore, the immune system involves a wide range of inflammatory cascade reactions, which may be potential targets in the treatment of ALI caused by II/R [59]. In clinical trials, directly targeting TLR4 failed to show good efficacy, but targeting CIRP may be beneficial in the treatment of ALI and ARDS caused by II/R [60].

## 3. Biomarkers for Alleviating II/R-Induced ALI

### 3.1. Nrf2 Signaling Pathway

Nuclear factor erythroid 2-related factor (Nrf2) is a key regulator of intracellular oxidative homeostasis and plays an important role in inflammatory defense response [61]. Previous studies have shown that Nrf2 plays a protective role in ALI induced by II/R [62]. After II/R, the conformation of Nrf2 complex changes. Nrf2 dissociates from Keap1 and enters the nucleus through translocation, where it combines with antioxidant response elements to induce anti-inflammatory and antioxidative effects and promote cell survival [63].

Inflammation plays an important role in the pathogenesis of ALI induced by II/R. Blocking the TLR/MyD88 pathway has been shown to reduce lung injury in mice [32]. II/R can upregulate the expression of TLR4 and Nrf2 in the lung tissue of mice, while Nrf2 enters the nucleus to regulate the expression of TLR4, reduce the release of inflammatory mediators, and alleviate ALI. Nrf2 induces the expression of heme oxygenase-1 (HO-1), which is related to the PI3K/Akt pathway. The Nrf2/TLR4/Akt pathway, thus, plays an important role in II/R-induced ALI and provides a new therapeutic target for the treatment of ALI [64].

**Figure 6:**
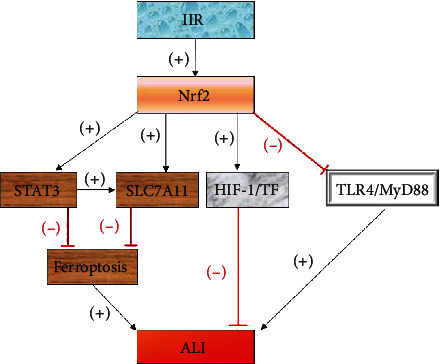
The Nrf2 pathway relieves ALI by regulating STAT3 and SLC7A11 to inhibit ferroptosis, by promoting HIF-1/TF and inhibiting TLR4.

A number of studies have shown that II/R injury involves a nonapoptotic pathway, and ferroptosis is an iron-dependent and caspase-independent type of nonapoptotic cell death [65]. Ferroptosis is different from classical apoptosis in that iron catalyzes the formation of lipid-free radicals and the depletion of glutathione (GSH) [66]. Downstream factors of Nrf2 (HO-1, glutathione peroxidase, and SLC7A11) play a crucial role in cell defense [67]. Activated STAT3 reduces apoptosis under ferroptosis and plays an important role in the inflammatory response and development of tumors [68]. Nrf2 and STAT3 are both antioxidant response elements. When cells are under oxidative stress, they can promote the expression of downstream target genes (such as SLC7A11) and reduce cell apoptosis and tissue damage. During II/R, Nrf2 and STAT3 jointly upregulate the expression of SLC7A11 and HO-1 and inhibit ferroptosis, thus reducing II/R-induced ALI and providing a new scheme for targeting the Nrf2 pathway in the treatment of II/R-induced ALI [69]. Similarly, p53 inhibitor IASPP inhibits ferroptosis through the Nrf2/HIF-1/TF signaling pathway [70]. Intestinal ischemic postconditioning promotes HO-1 expression through the Nrf2 pathway, inhibits oxidative stress and the inflammatory response, and reduces lung injury [71]. These studies all show that the Nrf2 pathway is an effective target for the treatment of ALI induced by II/R (Figure 6).

### 3.2. SIRT1 Signaling Pathway

SIRT1 is a nicotinamide adenine dinucleotide- (NAD-) dependent deacetylase. Through deacetylation of FOXO3, SIRT1 plays important antioxidative, anti-inflammatory, and antiapoptotic roles in ALI induced by II/R [72]. SIRT1 enhances cell viability under stress by regulating different downstream factors (p53, NF-*κ*B, and FOXO); for example, SIRT1 deacetylates FOXO3 and enhances cell resistance to oxidative stress through an antioxidant molecule (MnSOD) [73]. It is well known that the weakening of antioxidant stress and the activation of apoptotic signals play an important role in the pathogenesis of ALI induced by II/R. The antioxidant molecule MnSOD and antiapoptotic molecule Bcl-2 are closely related to ALI induced by II/R [74]. II/R activates a cascading reaction, which leads to the accumulation of reactive oxygen species, a decrease in SIRT1 and FOXO3 deacetylation, and a decrease in MnSOD and Bcl-2. Icariin can upregulate the expression of SIRT1, promote FOXO3 deacetylation, enhance the release of antioxidant molecules and antiapoptotic factors, and reduce the release of inflammatory mediators, which can protect lung tissue from injury caused by II/R; in contrast, the SIRT1/FOXO3 signaling pathway has little effect on experimental lung injury. The protective effect against ALI induced by II/R may be achieved by upregulating MnSOD and enhancing the expression of Bcl-2 [75].

**Figure 7:**
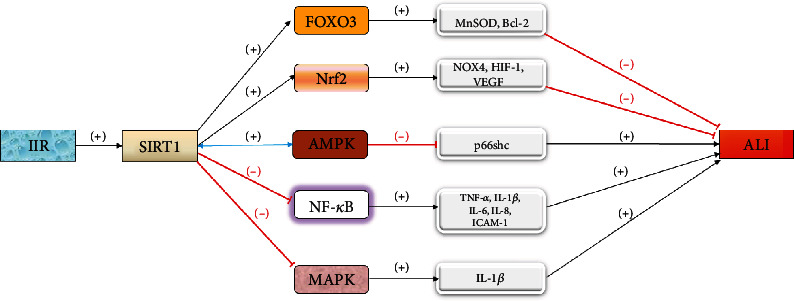
The SIRT1 pathway promotes FOXO3, Nrf2, and AMPK and inhibits NF-*κ*B and MAPK to alleviate ALI.

In addition, upregulation of SIRT1 level can increase the translocation of Nrf2, thereby inhibiting the activation of MAPK and protecting cells from damage [76]. Nrf2 is an important transcription factor, which not only protects cells from injury but also participates in ischemic angiogenesis [77]. Nrf2 regulates the expression of nonphagocytic oxidase 4 (NOX4), hypoxia-inducible factor 1A, and vascular endothelial growth factor and combines with them to resist oxidative stress [78]. In the model of II/R injury in mice, it was found that Nrf2 participates in the vascular remodeling caused by ALI after II/R injury. The increase in SIRT1 level induces the upregulation of Nrf2 and then promotes the angiogenesis of human pulmonary microvascular endothelial cells through gene regulation mediated by NOX4 [79]. Adenosine 5′-monophosphate- (AMP-) activated protein kinase (AMPK) is a key enzyme in bioenergy metabolism, which can be activated by a variety of anti-inflammatory drugs, and when coupled with SIRTs increases the activity of SIRT1 [80]. U-3 polyunsaturated fatty acids inhibit the release of inflammatory mediators and reduce ALI by activating the AMPK/SIRT1 pathway; in addition, these polyunsaturated fatty acids can inhibit p66Shc, restore claudin5 expression, restore the alveolar-capillary barrier, and reduce apoptosis by activating the AMPK/SIRT1 pathway. Therefore, regulating the AMPK/SIRT1 pathway may become a new mechanism to protect against ALI induced by II/R [81] (Figure 7).

## 4. Conclusion

**Figure 8:**
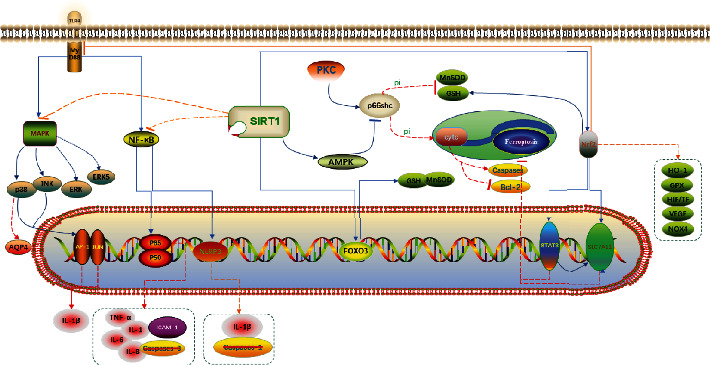
Cellular signal transduction pathways involved in ALI induced by II/R.

II/R injury is a common type of tissue and organ injury, secondary to intestinal and mesenteric vascular diseases. Ischemia leads to hypoxia, cell injury, and necrosis. However, the recovery of blood flow and oxygen during reperfusion results in bacterial translocation, tissue damage, inflammatory response, and oxidative stress. II/R can also lead to distal tissue damage and distal organ failure, of which lung injury is the most common, which is also the cause of the high mortality resulting from II/R injury. Previous studies have shown that II/R-induced ALI involves bacterial translocation, inflammatory response, oxidative stress, cell apoptosis, and necrosis; signaling pathways and markers are summarized in Figure 8. Further research has identified biomarkers that play an important role in the pathogenesis of II/R-induced ALI, which involves various targets and signaling pathways in the cascading reaction of II/R-induced ALI. By blocking pathways that can aggravate the disease and activating the pathways that can alleviate the disease, clinical trials have shown exciting results and suggest new approaches for better diagnosis and treatment of ALI caused by II/R. We believe that the continuing development of biomarkers will lead to novel therapeutic applications and cures for ALI caused by II/R.

## Data Availability

No data were used to support this study.
